# Evaluating the Alliance against Violence and Adversity Online Training Program: an online curriculum to support children, women, and gender-diverse people

**DOI:** 10.3389/fpubh.2026.1838043

**Published:** 2026-07-02

**Authors:** Stefan Kurbatfinski, Nicole Letourneau, Alexandria Lozowchuk, Josh Flis, Carrie Pohl, Andrea J. Deane, Ashley Stewart-Tufescu, Sarah Yercich, Alyson Campbell, Kharah Ross

**Affiliations:** 1 Alberta Children’s Hospital Research Institute, Calgary, AB, Canada; 2 Cumming School of Medicine, University of Calgary, Calgary, AB, Canada; 3 Faculty of Nursing, University of Calgary, Calgary, AB, Canada; 4 Hotchkiss Brain Institute, University of Calgary, Calgary, AB, Canada; 5 Faculty of Humanities and Social Sciences, Athabasca University, Athabasca, AB, Canada; 6 Faculty of Social Work, University of Manitoba, Winnipeg, MB, Canada; 7 Children’s Hospital Research Institute of Manitoba, University of Manitoba, Winnipeg, MB, Canada; 8 School of Criminology, Simon Fraser University, Burnaby, BC, Canada; 9 Department of Psychology, University of Calgary, Calgary, AB, Canada

**Keywords:** adverse childhood experiences, Alliance against Violence and Adversity (AVA), AVA Online, equity, diversity, inclusion, and accessibility, gender-based violence, implementation science, process evaluation

## Abstract

**Background:**

Gender-based violence (GBV) and adverse childhood experiences (ACEs) are Canadian public health concerns and human rights violations that inequitably impact the health of children, women, and gender-diverse people. The Alliance against Violence and Adversity (AVA) developed an Online Training Platform (AVA Online) to facilitate learning about GBV, ACEs, implementation science, and equity, diversity, inclusion, and accessibility for students, academics, and community partners. This study evaluated the first iteration of AVA Online using mixed-method survey data from platform users.

**Methods:**

AVA Online was designed to be suitable for diverse community and academic audiences, adaptable, scalable, and sustainable. Launched and live from February to December 2024, the following data were collected: (1) AVA Online registrant demographics, (2) module enrolment, and (3) survey feedback on content usefulness, applicability, novelty, and accessibility. Descriptive statistics, independent samples *t*-tests, and one-way ANOVAs were used to analyze quantitative data. Qualitative data were examined using a qualitative descriptive conventional content analysis approach.

**Results:**

A total of 74 content feedback responses were obtained. Most users were satisfied with accessibility (81%) and rated content as useful (mean score of 3.23/4), applicable (3.29/4), and novel (3.35/4). Areas for improvement included accessibility, curriculum streamlining, and platform navigation.

**Conclusion:**

AVA Online is a feasible cross-sector GBV and ACEs training platform with strong perceived usefulness and applicability, although areas for improvement were identified.

## 1 Introduction

Gender-based violence (GBV), defined as violence directed toward any individual due to their perceived or actual gender, is a pervasive form of harm that affects individuals worldwide (1–3). While GBV can affect anyone, girls, women, and gender-diverse people are disproportionately impacted, attributable to gender-specific vulnerabilities (1–3). Although GBV is usually conceptualized in the context of partners, such as those engaging in casual or committed sexual or romantic intimacies, family members (e.g., siblings, parents and their children), friends, and broader community members (e.g., co-workers, clergy, teachers) can all engage in GBV (1–3). Closely linked, adverse childhood experiences [ACEs; an umbrella term capturing the experiences of child abuse and neglect (4)], including physical, sexual, and emotional abuse, physical and emotional neglect, parental mental health concerns, parental loss, divorce, or separation, and parental incarceration, stem from the vulnerability and dependency that children have on adults (5). Both GBV and ACEs are known to undermine quality of life (6, 7).

Despite diverse prevention and intervention efforts, GBV and ACEs remain major public health concerns and human rights violations globally and within Canada (1, 2, 4, 8, 9). This is likely attributable to a knowledge-to-practice gap, whereby knowledge generated within academic settings does not consistently translate into accessible, actionable tools for community-based agencies positioned to respond (10–13). To address this gap, the Alliance against Violence and Adversity (AVA) developed AVA Online, an interdisciplinary training platform designed to strengthen community capacity relative to GBV and ACEs prevention and response through accessible, evidence-informed online learning (14).

Although GBV and ACEs are global issues (4), evidence also points to high levels in Canada. In 2018, 44% of Canadian women reported experiencing intimate partner violence after 15 years of age, a figure that underestimates the broader scope of GBV and disproportionately affects younger women, Indigenous women, women with disabilities, and sexual and gender minority individuals (15). At its most extreme, GBV results in homicide; it is estimated that one woman or girl is murdered every 48 h in Canada (16). Similarly, 36% of Canadians aged 45–85 report exposure to at least two ACEs, with higher prevalence among women, sexual and gender minorities, and certain provinces (17). Male children tend to experience more physical abuse, while female children are more likely to experience emotional neglect, be exposed to sexual and emotional abuse, and reside in a household with an individual experiencing a mental health concern (17). Evidently, GBV and ACEs remain prevalent and socially patterned in Canada despite extensive social protection systems (1, 2, 9, 18).

GBV and ACEs are closely intertwined and rarely occur in isolation. They cluster, remain underreported, and are chronic stressors that can “get under the skin” in ways that increase risk for lifelong disease, poverty, and intergenerational social disparities (19–21). Exposure to ACEs is associated with both the experience and use of GBV, with stronger effects among racialized, disabled, and sexual and gender minority groups, whose intersecting identities shape heightened vulnerability within systems of oppression (17, 22). Individuals exposed to ACEs and/or GBV are more likely to report experiencing mental (e.g., anxiety, depression) and physical (e.g., cardiovascular challenges) health sequelae (23, 24), suicidal ideation (25, 26), and lower socioeconomic attainment (27, 28). Empirical evidence reveals lower life expectancy among individuals exposed to GBV (16) and ACEs (29).

GBV and ACEs also impose substantial economic burdens. In Canada, GBV has been estimated to cost $7.4 billion annually (Government of Canada, 2012), while in North America, ACEs are estimated to cost approximately $748 billion (21). These figures are likely conservative due to underreporting and outdated estimates. Addressing GBV and ACEs therefore has implications not only for individual health and wellbeing, but also for labor market participation, economic productivity, and broader societal stability.

Persistent gaps remain in translating evidence on GBV and ACEs into practice, prevention, and policy settings (10–13). Academics often work in competitive environments with inflexible deadlines and specific research agendas, affecting their engagement with others outside their direct research team (30, 31). Community-based agencies providing direct support to individuals affected by GBV and ACEs often lack access to, knowledge of, or the capacity to develop, evaluate, and implement relevant evidence-based practices (32–38). Although the knowledge necessary to prevent or address GBV and ACEs may exist, it is not effectively reaching those who address or those affected by GBV or ACEs (13).

A common way to address the knowledge-to-practice gap is by creating curriculum or training resources intended to disseminate research to practitioners and community users (39–41). Several well regarded GBV and/or ACEs online learning platforms have demonstrated effectiveness in increasing awareness, knowledge, and confidence among researchers, service providers, and trainees, including VEGA (Violence, Evidence, Guidance, Action) (42), VetoViolence (43), Violence Prevention in Practice (44), Zero Abuse Project (45), and ACEs Aware (46). These initiatives have made important contributions by equipping learners to recognize violence, respond to disclosures, and apply trauma- and violence-informed principles in individual interactions. Despite these successes, there is evidence of several challenges that continue to affect initiatives of this nature and may decrease the sustained effectiveness of online curricula overall (39–41). These challenges include: (1) not being implemented as an iterative, growing platform (i.e., not sufficiently scalable or sustainable); (2) designed for a specific subset of users (i.e., audience too narrow to be broadly impactful); (3) little interdisciplinary or cross-sector collaboration or co-design; (4) lack of contextual applicability; and (5) inconsistent use of theory or approach to guide development (39). The Knowledge-to-Action framework is a process developed by Graham and colleagues to address these limitations by bridging the divide between knowledge produced by academics and the care provided within community-based organizations (10). The framework conceptualizes knowledge translation as an iterative process including knowledge creation (i.e., inquiry, synthesis, tools/products) and action (i.e., implementation, evaluation) to improve accessibility and sustainability of evidence-informed practices (10). Since knowledge is continuously evolving, the framework emphasizes ongoing knowledge creation and action, reflecting a cyclical approach to knowledge mobilization (10). Knowledge-to-Action provides a useful framework for addressing implementation challenges and highlights the importance of iterative refinement processes within online training platforms and curricula to enhance usefulness, accessibility, and practical application of knowledge.

AVA is a pan-Canadian research and training initiative funded by the Canadian Institutes of Health Research (CIHR) whose core aim is to strengthen GBV and ACEs prevention and response by narrowing the longstanding knowledge-to-practice gaps (14). AVA was established to bring together researchers, service providers, trainees, policymakers, and individuals with lived experience to collaboratively advance evidence-informed solutions to GBV and ACEs (14). AVA’s Health Research Training Platform integrates mentorship, early-career scholar support, implementation science training, and community-engaged internships to foster interdisciplinary, cross-sector capacity building to address GBV and ACEs in community. It consists of five interconnected programs, of which AVA Online is the most accessible and public facing. AVA Online was specifically designed as per the Knowledge-to-Action framework to address challenges that often affect online knowledge dissemination curricula (14, 47).

Launched in February 2024, AVA Online is a free, asynchronous, and self-paced curriculum addressing GBV, ACEs, equity, diversity, inclusion, and accessibility, and implementation science. Designed as a cross-sector training platform, it aims to support shared language, conceptual alignment, and implementation readiness among researchers, trainees, and community-based practitioners. By embedding implementation science principles within a flexible digital learning environment, AVA Online represents an effort to translate evidence into practice at scale within a geographically and jurisdictionally diverse national context, with an emphasis on applied learning, cross-sector (community) engagement, and co-creation. Despite growing investment in digital training platforms (43–46), limited empirical research has effectively evaluated engagement, accessibility, and impacts. Examining AVA Online as a national case therefore provides insight into the opportunities and constraints of online, interdisciplinary capacity-building initiatives within the GBV and ACEs prevention field.

The purpose of this process evaluation was to evaluate the first iteration of the AVA Online training program. Specifically, the evaluation focused on AVA user sociodemographic and professional backgrounds, user engagement or registration with AVA Online content, module feedback that assessed the usefulness, applicability, and novelty of the AVA Online modules, content accessibility, and to identify opportunities to improve the program. This study provides unique contributions by moving beyond satisfaction metrics to identify practical and structural factors that shape how cross-sector digital training platforms facilitate, and potentially constrain, knowledge-to-practice in GBV and ACEs prevention contexts.

## 2 Methods

This study used mixed-methods data to evaluate AVA Online. The methods were reviewed by a University of Calgary Research Ethics Board (REB) Officer and deemed exempt from formal ethics review as data are primarily collected for program evaluation purposes. Nonetheless, best ethical standards were observed, with AVA users being informed of possible data collection and its purpose upon registration. Only aggregate or de-identified user registration or module completion data were used. Users were directed to an optional feedback survey link after completing an AVA module and any module feedback was provided anonymously.

### 2.1 Participants

Participants were users who accessed AVA Online content between February 12 and December 31, 2024. AVA Online evaluation data were obtained from three sources with overlapping user or participant pools. Due to anonymity and confidentiality reasons, it was not possible to link participant data across data sources.

First was the AVA Online registration data (Data Source #1). AVA Online was hosted on the Raising Interdisciplinary Scientists Excellence (RISE) learning management system, built to host several CIHR-funded health research training platform curricula, of which AVA Online is one. To access content, users first have to register with one of the health research training platform curricula. Users who wanted to access content through AVA registered on RISE with AVA Online specifically. A total of 118 individuals registered with AVA Online.

Second was module enrollment data (Data Source #2). RISE also tracked enrollment for each module, or discrete training unit within AVA Online. Because the original intention was for each CIHR-funded health research training platform to make their content openly available to users from other platforms, AVA Online content was open to all users registered on RISE, regardless of whether they accessed RISE through AVA Online (Data Source #1 above) or another separate platform (data not accessible).

Third was module feedback survey data (Data Source #3). All users who completed AVA Online modules (Data Source #2 above, which consists of AVA registrants (Data Source #1) and other training platform users) were invited to provide voluntary and anonymous feedback after completing each AVA Online module. A total of 74 module feedback responses were received.

### 2.2 AVA Online development

AVA Online’s design was led by the AVA Online Chair (KMR) based on the original program described in the AVA grant proposal (Principal Investigator: NL). First, AVA Online was created as per AVA’s guiding principles and aims and program requirements. As a result, AVA Online content needed to align with the four core AVA topic areas: adult and child experiences of GBV and ACEs, implementation science, and equity, diversity, inclusion, and accessibility principles (14). AVA Online also needed to complement and support other AVA programming (e.g., Community Agency Internship Program; 49) and knowledge dissemination activities.

Second, to meet goals related to design in accordance with open and flexible adult learning principles, approaches successfully used by Athabasca University, a Canadian open, online university that specializes in flexible adult learning (48), were also incorporated. The program needed to be deliverable online and maximize learning flexibility (i.e., continuous and asynchronous enrollment and self-paced learning), in order to be accessible to busy community-based professionals with full-time jobs and trainees enrolled in full-time programs. Also, the program and content needed to be accessible for diverse learners (e.g., hearing or vision impaired) and available in both official Canadian languages (French and English).

Third, design approach was guided by the Knowledge-to-Action framework (10); content was designed to be adaptable, scalable, and sustainable (49). Modules could be added or modified as evidence evolved, structured to support expansion for specific professional or cultural contexts, and maintained with minimal ongoing staffing or financial requirements.

An interdisciplinary and cross-sector co-design approach incorporating multiple perspectives, contexts and cultures was specifically used, with program review and feedback provided by several advisory groups (i.e., AVA Leadership Team, the AVA Platform Advisory Committee, the AVA Online Working Group) that included trainees, academics, community practitioners, and equity, diversity, inclusion, and accessibility champions. The program proposal (including the evaluation strategy) was iteratively revised and reviewed until consensus was reached. Once the program framework was completed, content and materials were solicited from AVA members, including academic and community partners. To be included, content had to align with AVA topic areas, adhere to open and asynchronous adult learning principles, be available in English or French, include a mechanism for proof of completion, and support long-term sustainability. Content gaps were noted and addressed by approaching academic or community experts for new materials. Proof of completion (i.e., certificates, quizzes) were imported from existing materials if available or developed. The program structure and content were then built into the RISE learning management system (50), a Moodle-based platform supporting multiple health research training initiatives.

#### 2.2.1 AVA Online structure

**Figure 1:**
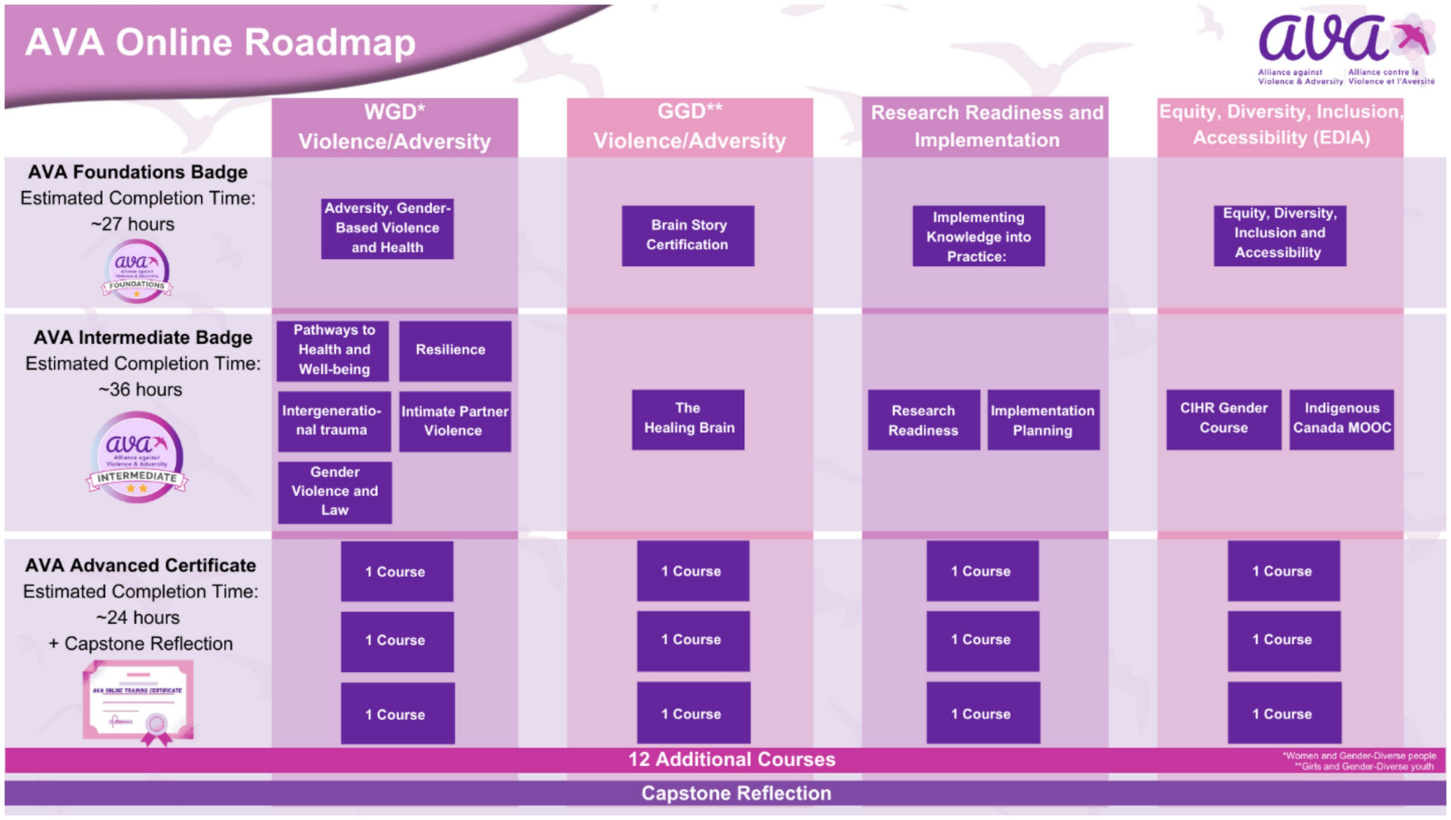
AVA Online roadmap.

The AVA Online curriculum road map is presented in Figure 1. Discrete units of content and materials are called “modules.” Modules are organized into core pillars, levels, and streams. “Pillars” refers to core AVA topic areas, specifically adult GBV and ACEs, child GBV and ACEs, implementation science, and equity, diversity, inclusion, and accessibility. “Levels” refers to advancing micro-credentialing, moving from basic to advanced information: AVA Foundations, Intermediate, and Advanced. AVA Foundations and Intermediate include stable foundational content, updated as necessary. In contrast, AVA Advanced builds on these materials and remains flexible, with new modules added as content becomes available. “Streams” refers to content that is curated for specific audiences or purposes. For example, there is an AVA Triadic Mentorship Program or Community Agency Internship Program “stream” that includes additional content on mentorship and professional development. Additional “streams” can be designed for specific professions (i.e., first responders, lawyers, social workers), youth, and to allow use of AVA Online as an e-text resource as part of undergraduate courses.

Proof of module completion is demonstrated through submission of a certificate or transcript, or by passing an AVA-hosted quiz. Modules may be delivered internally within the AVA Online learning management system, externally through partner platforms, or in a mixed format in which external content is paired with internal evaluation. When external materials do not include proof of completion, users complete an AVA-hosted quiz to document engagement.

#### 2.2.2 Credentialing and completion requirements

Users may complete AVA Online through structured micro-credential pathways (“streams”) or through individual module selection (a pick-your-own-adventure approach). If users chose to complete AVA Online content through structured pathways, three micro-credentialed levels are available: Foundations, Intermediate, and the AVA Certificate. Completion of Foundations or Intermediate awards a badge, while the AVA Certificate requires completion of Advanced modules and submission of a Capstone Reflection assignment. The Capstone invites users to reflect on their learning journey and its application to professional or academic practice and is assessed for completion rather than content quality. The AVA Online Certificate was also designed to be comparable to a 3-credit university course and to potentially be used for professional development education.

### 2.3 Data collection

As described above, AVA Online evaluation data were obtained from three sources: AVA Online user registration data, module enrollment data, and module feedback survey data.

#### 2.3.1 AVA Online user registration

Upon registration with AVA Online through RISE, users were asked to provide sociodemographic and professional background information. Specifically, users self-reported their sex and gender, race/ethnicity, geographic location, and professional role. Professional roles were trainees (students), independent researchers, community practitioners, community staff members, research staff, or members of the public.

#### 2.3.2 AVA Online module enrollment

RISE tracks registration per each AVA Online module. Registration data for each AVA Online module was extracted to assess overall user engagement with each unit of content.

#### 2.3.3 AVA Online feedback survey

Module feedback questions (English and French) were embedded at the end of each module. Feedback was collected through the RISE platform from February to August 2024; beginning in September 2024, the same feedback items were administered via SurveyMonkey due to data export limitations in the RISE platform. Responses from both platforms were combined for analysis. Item wording and response options were unchanged across platforms.

Module feedback surveys consisted of four sections of questions: respondent background information; usefulness, applicability and novelty of the AVA Online modules; suggestions for improvement; and module accessibility.

##### 2.3.3.1 Respondent background information

Respondents provided sociodemographic and professional background characteristics (i.e., sex/gender, race/ethnicity, geographic location, and professional role). This information was used to characterize respondents.

##### 2.3.3.2 Module usefulness, applicability and novelty

Respondents completed questions evaluating perceived usefulness (“how useful was the course content?”), applicability to practice, work or training program (“will you be able to apply what you learned to your practice, work or training?”), and content novelty (“did you learn something new?”) for each module. Responses were captured using a 5-point Likert scale, ranging from “Not at all” (0) to “A lot” (4).

##### 2.3.3.3 Suggestions for improvement

Two open-ended questions were used to obtain unstructured feedback on each module. Specifically, respondents were asked “what did you like about the module?” (positive feedback) and “what could we do to improve the module?” (opportunities for improvement).

##### 2.3.3.4 Module accessibility satisfaction

Respondents were also asked if module accessibility could be improved (yes/no). If a respondent indicated “yes,” an open-ended question prompted “please tell us how we can improve module content accessibility.”

### 2.4 Analytic approach

Data on AVA Online user registration, module enrollment, and module feedback were downloaded and separately analyzed. All quantitative data were analyzed using IBM SPSS v.28 (IBM, 2021). Summary statistics [e.g., means, standard deviations (SD)] were generated to characterize background or descriptive information. Group differences were tested using independent samples *t*-tests (comparing two means) or one-way ANOVAs (comparing more than two means). For significant one-way ANOVAs, *post-hoc* tests using Fisher’s Least Significant Differences (LSD) were run to test for significant group differences. Quantitative comparisons were exploratory and examined differences in ratings by module level and respondent role where cell sizes allowed.

For qualitative, open-ended responses were analyzed using a qualitative descriptive conventional content analysis approach (51), an approach that is appropriate for exploratory research with limited theory. Categories are not predefined but are inductively derived from the data (51, 52). Given that most responses were short (e.g., a few words or sentences), a manifest-level approach was used to capture surface meaning, resulting in a systematic but minimally interpretive summary of participant perspectives. Data were stored and analyses conducted in a Word document by one author (KMR), similar to methods described elsewhere (47). Text responses were synthesized by identifying similarities and differences between the responses, separately for each open-ended question: (1) what was liked about the content and (2) opportunities for improvement. For each set of question responses, similar concepts were grouped into categories that reflected similar ideas. Concept categories were supported using direct quote examples from respondents, and labeled by Participant ID.

## 3 Results

### 3.1 AVA Online users

A total of 118 users were registered with AVA Online. User characteristics are summarized in Table 1. There was even distribution with respect to students or trainees, community practitioners or staff, and research staff, with independent researchers being the least represented (12%). Most users identified as a woman (49%), female (52%), and white (35%). Although most users were in Canada, registrants also came from China, Ireland, Nigeria, and the United States. Of the users located in Canada, most were from Alberta (29%) and Ontario (14%). Of note, only Newfoundland, the Yukon, and Nunavut were not represented.

**Table 1:** AVA Online user characteristics.

Variable	Specific group	Mean ± SD or % (N)	
Study variable		AVA user registration	Module feedback respondents
Role	Trainee or student	25% (29)	52% (38)
	Independent researcher	12% (14)	26% (19)
	Community practitioner or staff	19% (23)	12% (9)
	Research staff	18% (21)	11% (8)
	Prefer not to say	26% (31)	–
Gender	Non-binary	2% (2)	1% (1)
	Woman	49% (58)	75% (55)
	Man	5% (6)	23% (17)
	Prefer not to say	44% (52)	1% (1)
Sex	Female	52% (61)	75% (55)
	Male	5% (6)	25% (18)
	Prefer not to say	43% (51)	1% (1)
Race/ethnicity	White	35% (41)	55% (41)
	Black	1% (1)	3% (2)
	Chinese	2% (2)	4% (3)
	South Asian	8% (9)	22% (16)
	Other race/ethnicity	8% (8)	0% (0)
	Prefer not to say	48% (57)	16% (12)
Country	Canada	97% (114)	–
	United States	1% (1)	–
	China	1% (1)	–
	Ireland	1% (1)	–
	Nigeria	1% (1)	–
Province	British Columbia	8% (9)	20% (15)
	Alberta	29% (34)	55% (41)
	Saskatchewan	1% (1)	–
	Manitoba	11% (13)	1% (1)
	Ontario	14% (16)	20% (15)
	Quebec	5% (6)	–
	New Brunswick	7% (8)	3% (2)
	Prince Edward Island	2% (2)	–
	Nova Scotia	1% (1)	–
	Northwest Territories	1% (1)	–
	Preferred not to say	23% (27)	–
AVA registrations per module		3.61 ± 6.92	–

### 3.2 AVA Online module registration

At time of evaluation, AVA Online included 47 modules (four within AVA Foundations, 21 within AVA Intermediate, and 22 within AVA Advanced). On average, users had registered in 3.61 ± 6.92 modules (mean = 2.00, range 1–51). More users had registered in AVA Foundations modules (mean = 162, SD = 5.29), compared to AVA Intermediate (mean = 6.20, SD = 0.422) and Advanced (mean = 4.06, SD = 0.348).

### 3.3 AVA Online module feedback

Seventy-four feedback responses were available for AVA Foundations and AVA Intermediate modules. Respondent background information is reported in Table 1. Respondents were mostly trainees or students (52%), primarily identified as cisgender female (75%), and white (55%) or South Asian (22%). Most resided in Alberta (55%), British Columbia (20%), or Ontario (20%).

#### 3.3.1 AVA Online feedback: usefulness, applicability, and novelty

A total of 74 module feedback surveys were completed. Module summaries are reported in Table 2. Overall, across all modules, content was rated highly useful, applicable, and novel (i.e., ≥ 4, “a lot”). Most responses were provided for AVA Foundation modules (75%). Separate independent samples *t*-tests were used to assess mean differences in usefulness, applicability, and novelty between AVA Foundations and AVA Intermediate levels. Given that there could be systematic differences in groups (i.e., Foundation verses Intermediate levels) by content and difficulty that could affect variance homogeneity, *t*-tests for which equal variances are not assumed were reported (note that pattern of results did not vary by equal variance assumptions). No differences by module level were detected for perceived usefulness [*t*(29) = 1.01, *p* = 0.323, Cohen’s *d* = 0.295, 0.95 CI (−0.213, 0.802)] or novelty of content [*t*(40) = −1.01, *p* = 0.321, Cohen’s *d* = 0.649, 0.95 CI (−0.763, 0.272)]. However, differences were detected for applicability to practice [*t*(28) = 2.13, *p* = 0.042, Cohen’s *d* = 0.649, 0.95 CI (0.129, 1.17)], such that AVA Intermediate modules were rated as slightly less applicable to practice (mean = 2.86, SD = 1.20) than the Foundations-level modules (mean = 3.46, SD = 0.803).

**Table 2:** Module summaries.

Module	% (N)	Useful	Applicability	Novel
Total^a^	100% (74)	3.23 ± 0.798	3.29 ± 0.964	3.35 ± 0.858
AVA foundations^b^	72% (53)	3.28 ± 0.717	3.46 ± 0.803	3.29 ± 0.893
Brain story	12% (9)	3.78 ± 0.441	3.75 ± 0.463	3.87 ± 0.893
Equity, diversity, inclusion, and accessibility	20% (15)	2.80 ± 0.775	3.27 ± 0.884	2.53 ± 0.990
Implementation science	15% (11)	3.45 ± 0.688	3.36 ± 0.924	3.73 ± 0.467
Introduction to gender-based violence	24% (18)	3.33 ± 0.594	3.56 ± 0.784	3.39 ± 0.778
AVA intermediate^c^	28% (21)	3.05 ± 0.973	2.86 ± 1.20	3.50 ± 0.761

^a^All modules across all levels (*n* = 74). ^b^AVA Foundation modules only (*n* = 4). ^c^AVA Intermediate modules only (*n* = 21).

Ratings per each AVA Foundations module are presented in Table 2. Three one-way ANOVAs were used to separately test mean differences in usefulness, applicability, and novelty among the four AVA Foundations modules only. Each module was rated as highly applicable, with no differences by module [*F*(3, 48) = 0.764, *p* = 0.520]. Differences did emerge between the modules with respect to perceived usefulness [*F*(3, 49) = 4.80, *p* = 0.005] and novelty [*F*(3, 48) = 4.54, *p* < 0.001]. *Post-hoc* tests revealed that, while most modules were consistently rated as highly useful and novel, participant feedback on the Introduction to Equity, Diversity, Inclusion, and Accessibility module was more reserved (Table 2). AVA Intermediate module response rates were between one and three (median = 2) per module; sample size is not sufficiently large to analyze responses per module.

#### 3.3.2 Positive feedback

A total of 56 responses were provided in response to the question about what was liked about module content and approach. Responses fell into three categories, summarized below, relating to content (1) quality, (2) applicability, and (3) novelty. Consistent with the overall positive feedback, one respondent indicated an eagerness to pursue the AVA micro-credentialing: “I want to attain my AVA Intermediate badge” (ID 5).

##### 3.3.2.1 Content quality

The first category that emerged was labeled “content quality” and was defined by responses (*n* = 26) that expressed appreciation for the overall quality of the materials (e.g., “this course was awesome,” ID 7). This included remarks on the quality of the content delivery approach (“The Video and PDF slide deck were both great,” ID 43; “I appreciated the opportunity to work at my own pace,” ID 13), the quality of content organization (“The course was well-organized and followed a logical sequence,” ID 46), and quality of supporting materials (“Provided many references and resources,” ID 30; “The ability to download the participant guide for future reference is wonderful,” ID 48).

##### 3.3.2.2 Content applicability

The second category that emerged was labeled “content applicability” and was defined by responses (*n* = 12) that expressed appreciation for the emphasis on useful or applied knowledge within module content. This included perceived applicability for community and healthcare practitioners, parents, educators (“This course is great for practitioners, educators or parents,” ID 48; “…*better equip me to thrive in interactions with young learners*,” ID 13; “…*making my class space a better environment for my Indigenous students*,” ID 8). Applicability came from content that incorporated intersectional perspectives (“intersectional lens is… huge and sometimes overlooked aspect of this work,” ID 1) and a focus on providing actionable, evidence-based information (“I liked that it was evidence-based,” ID 39; “it included prevention and intervention, and statistics to back up the information,” ID 52; “I liked the combination of statistics with impact statements,” ID 1).

##### 3.3.2.3 Content novelty

The third category was labeled “content novelty,” reflecting responses (*n* = 10) that indicated the information was new, valuable and potentially changed the respondent’s perspectives. For example, some responses spoke to being surprised by the scope and impact of GBV and ACEs (“It was eye-opening to see how (gender-based violence) is still a significant issue,” ID 11; *“*Staggering statistics that really bring forth the scale of gender-based violence and its consequences,” ID 34). Other perspectives spoke to learning new approaches to practice (“I learned the terminology for many harmful practices that I knew existed but didn’t know were having these specific terms,” ID 44) and factors that could protect against or buffer the impact of GBV and ACEs (“… Really insightful to see how effective partner support could be,” ID 16).

#### 3.3.3 Opportunities for improvement

A total of 51 responses were provided to the question about what could be improved with respect to module content and approach. Responses fell into four categories. The first was a simple category where respondents indicated they had no suggestions for improvement (*n* = 29; “nothing really to improve,” ID 9). Other categories were recommendations for module content improvement, curriculum improvement, and learning platform improvement.

##### 3.3.3.1 Module content improvement

The second category was labeled “module content improvement,” defined by responses (*n* = 24) that made specific recommendations for improving modules. The second category included six sub-categories. The first sub-category (*n* = 6) related to suggestions for improving content organization (“agenda/outline for content,” ID 49). Respondents suggested that longer content be broken up with opportunities for engagement and reflection interspersed throughout (“…*to have some opportunity within the video to pause and reflect on the material*,” ID 37; “several shorter videos with questions in-between would be more engaging,” ID 41). The second sub-category (*n* = 4) related to suggestions to make content more generalizable by including more real-life examples (“More case study sections related to other life scenarios,” ID 32) or more diverse contexts or perspectives (“It would have been better if the course was designed from the perspective of community agencies,” ID 42; “…*hard to reflect on because my work experience is not in a shelter setting*,” ID 48). The third sub-category (*n* = 3) was incorporating more closed captioning, subtitles, or transcripts (“*I think subtitles were my only concern*…,” ID 45); the fourth sub-category (*n* = 2) was related to checking that content is updated (“Some of the survey statistics were from the early 2000s,” ID 1); the fifth sub-category (*n* = 3) contained responses about quizzes (“make the quiz easier,” ID 36). The last sub-category was the only one that emerged for a specific and external module: Introduction to Equity, Diversity, Inclusion and Accessibility (*n* = 4). Respondents particularly noted issues with the module media (“It wasn’t obvious that you had to click to go to the next slide. Sometimes it felt as if I should be waiting for something or the screen was frozen,” ID 38; “Sometimes I found myself waiting for info when in actuality it was just a title slide or slide that went on for 10 seconds,” ID 40).

##### 3.3.3.2 Curriculum improvement

The third category was labeled “curriculum improvement,” defined by responses (*n* = 3) that suggested changes to the module order within the curriculum. In particular, it was suggested that the AVA Intermediate Healing Brain module should be completed before the AVA Foundations Health Brain module (“I felt this course was easier to digest than brain story. It almost feels like this should come before that,” ID 55).

##### 3.3.3.3 Learning platform improvement

The fourth category was labeled “learning platform improvement,” defined by responses that suggested improvements for the RISE platform (*n* = 2). This included issues navigating RISE (“improve how to access the courses on the RISE platform,” ID 5) and RISE not automatically marking modules as completed or awarding micro-credentials (“Minor issues with obtaining certificates. RISE platform not marking courses complete,” ID 33).

#### 3.3.4 Satisfaction with module accessibility

Most respondents (81%) selected “no” when asked if module accessibility could be improved. Five responses were provided that suggested ways to improve accessibility. Two categories emerged: (1) closed captioning or subtitles and (2) visual contrast.

##### 3.3.4.1 Closed captioning or subtitles

The first category was labeled “closed captioning or subtitles” (*n* = 3), defined by responses that indicated the need for more closed captions, subtitles, or transcripts available (“I’m a visual learner and would appreciate having transcripts available to read. This would also help those who are hard of hearing,” ID 59). Of note, each of these responses were for an external module, the Brain Story Certification.

##### 3.3.4.2 Visual contrast

The second category was labeled “visual contrast” (*n* = 2), defined by responses to improve accessibility for individuals with impaired eyesight. Specific suggestions were for “better slide contrast” (ID 46), making sure that font colors are readable (“…*font colors caused contrast issues*,” ID 11), and “having slightly bigger fonts” (ID 11) or the ability to enlarge fonts.

## 4 Discussion

The purpose of this process evaluation was to evaluate the first iteration of AVA Online, an open, accessible online training program addressing GBV and ACEs. Preliminary findings suggested that AVA Online’s content and approach are useful, applicable to practice, novel, and accessible to a wide audience. Findings also identified areas for improvement, including accessibility issues (primarily related to external content) and platform-specific usability challenges within the learning management system. The evaluation also provided direction for ongoing scaling and adaptation of AVA Online, supporting continued expansion of this work and its potential impact on GBV and ACEs. The following discussion elaborates on key findings, areas for improvement, opportunities for scaling and adaptation, and limitations.

### 4.1 Findings related to accessibility and implemented changes

AVA Online was designed for both community and academic audiences, intentionally integrating these groups within a single training platform rather than treating them as separate contexts or “cultures.” The overall success of this approach is reflected in the composition of users, evaluation feedback, and post-evaluation uptake, including recognition of AVA Online by community partners as a professional development opportunity (e.g., Alberta Association of Nurses) and consideration by universities for course credit (e.g., Athabasca University). Notably, although AVA Online was originally intended for a Canadian audience, users also reported locations outside of Canada (e.g., U. S., China, Ireland, Nigeria), suggesting broader global appeal and opportunities for expansion.

Despite this reach, no users appeared to reside in Nunavut, the Yukon, or Newfoundland. The Northwest Territories, Yukon, and parts of Newfoundland and Labrador, are geographically remote and sparsely populated regions of Canada (53). In addition, the territories are composed of predominantly Indigenous populations who have experienced racism, child welfare inequities, and other harms arising from historical and ongoing colonial systems (54). Because these regions are more isolated (54), the absence of uptake may reflect that AVA has not penetrated these areas to date. As highlighted in integrated frameworks on rural healthy equity, digital delivery may reduce geographic barriers, but equitable reach is not automatic (55). Accordingly, strategies such as relationship-building and trust development may be necessary to support engagement in structurally and geographically underserved contexts (56).

Designing a single platform for cross-sector audiences also presents both opportunities and tensions. While AVA Online prioritizes accessibility, preliminary feedback identified challenges that are being addressed in AVA Online 2.0. Some external modules were deemed less accessible than internally developed content (i.e., no closed captioning). Although online training programs can reduce certain accessibility barriers, they can fail to address important barriers or even introduce new ones, particularly for certain populations (57). For example, online training platforms can reduce but also inadvertently exacerbate accessibility inequities among individuals living with disabilities (58), who are also disproportionately affected by ACEs and/or GBV (59, 60). This is important to consider when online training platforms are being developed and refined; although addressing all accessibility-related challenges may not be feasible, efforts to minimize inequities among previously affected groups remain especially important.

Moreover, accessibility issues were also noted for the learning management system (i.e., difficult to navigate, not user friendly). These challenges highlight the importance of aligning platform selection with not only the intended purpose, but also the target audience (61). Even well-established learning management systems may not be suitable for delivering certain training modules, and high-quality content may be undermined by navigation and accessibility concerns (61–63). Ultimately, such issues may reduce user engagement and negatively affect learning outcomes (61–63). Accordingly, the selection of a learning management system that is both functional and accessible should be viewed as a critical component of the development and implementation of online training platforms, rather than a purely administrative decision (64).

It should also be noted that the automated feedback approach may represent another accessibility consideration. Automated quizzes were selected to reduce administrative burden and support scalability, consistent with approaches used in other online learning environments, including massive open online courses (65–67). However, standardized evaluation formats may present barriers for some learners. Although respondents did not identify this as a concern and assessments were intentionally designed to be low stakes (untimed with multiple attempts), ongoing monitoring of engagement and exploration of alternative assessment formats will remain important to ensure equitable use of AVA Online.

### 4.2 Findings related to perceived usefulness and impact and implemented changes

Most AVA Online users found the online platform to be useful and to support learning related to GBV and ACEs. Quantitative ratings demonstrated consistently high levels of perceived usefulness, applicability, and novelty across modules. These findings were reinforced by qualitative feedback which highlighted strong content quality, well-organized structure, and flexible delivery formats that supported self-paced learning. Respondents also emphasized the value of evidence-based content, particularly the integration of statistical information and downloadable resources. Collectively, these findings suggest that AVA Online was successful in achieving its primary aim of disseminating knowledge in an impactful way across diverse audience including trainees, practitioners, and researchers.

While overall ratings of AVA Online applicability were strong, quantitative data indicated slightly lower applicability for one Intermediate-level module. Although the difference was small, this finding is noteworthy given that community-based professionals, academics, and students often work in time-constrained environments (68). If content is perceived as less applicable, users may be less likely to complete or continue engaging with online training platforms (39–41, 69). Consistent with the guiding Knowledge-to-Action framework (69) and a commitment to continuous improvement, this module was replaced in AVA Online 2.0. Ongoing evaluation will assess whether the revised content improves user experience and perceived relevance.

### 4.3 Areas for improvements and implemented changes

Feedback from this evaluation directly informed the development of AVA Online 2.0, including revisions, replacement, and reorganization of select modules. Foundational and intermediate content was reorganized in response to user feedback to improve sequencing and progression. Alternative content was also identified for external modules that presented challenges in the first iteration. Feedback on external modules is being shared with content partners. Because accessibility issues were particularly evident in externally sourced content (e.g., lack of closed captioning), more specific accessibility criteria have now been integrated into inclusion and exclusion decisions for external materials. Lastly, since issues were noted with the original learning management system, AVA Online 2.0 was moved to a different platform offering improved navigation and accessibility features.

Findings from this evaluation highlighted other areas for ongoing improvement. First, there is a need for more case-based and practice-oriented scenarios, as well as content reflecting a broader range of professional roles. This aligns with broader literature indicating that the inclusion of case-based and practice-oriented scenarios within online training platforms may enhance engagement and support the application of knowledge by situating learners within authentic contexts resembling their real-world experiences and responsibilities (70). By fostering a stronger connection between the user and the content being delivered, online training platforms are anticipated to foster greater engagement (70).

Second, respondents suggested improving module content by breaking longer content into smaller sections and incorporating more opportunities for reflection. Third, greater attention to delivery quality was recommended by respondents, including more intuitive navigation and improved visual design (e.g., color contrast and font size).

Additional accessibility priorities include making content and branding more engaging for men and boys, who are considered essential to have meaningful impact on addressing GBV and ACEs (71). Also, given higher prevalence of GBV and ACEs in rural, remote, and northern regions of Canada (72), it is important to evaluate accessibility in these regions. Overall, these findings reinforce that digital training platforms are not a substitute for equity. For policy mandates to be effective, digital infrastructure must be paired with sustained funding for relational outreach and trust-building specifically tailored to reach geographically isolated and structurally underserved regions, such as those in Northern Canada. Further, for practitioners, technical accessibility (e.g., intuitive navigation, and closed captioning) is not a convenience but a fundamental prerequisite for successful mobilization of evidence into practice.

In contrast to other training platforms that do not systematically incorporate user feedback or use guiding frameworks (39), AVA’s use of the Knowledge-to-Action framework supports continuous improvement informed by direct user input. This is reflected in the implemented changes described above, which will continue to be evaluated to ensure AVA Online remains accessible, applicable, and useful for users.

### 4.4 Limitations

There are several methodological limitations to consider. First, challenges with data collection were discovered partway through the pilot evaluation. Specifically, the RISE platform was not able to provide summaries of user registration, module enrollment, completion data, or easily accessible data from feedback surveys. As a result, feedback surveys were transitioned to another platform (SurveyMonkey), and a co-author (JF) manually extracted and aggregated data from RISE. This potential limitation, in addition to user accessibility and navigation challenges with the learning management system, may partly explain the smaller sample size and low response rates (i.e., there were indications that some users discontinued engagement early in the curriculum).

Second, not all users provided feedback, and representativeness cannot be determined. It is not ethically permissible to require users to provide feedback as a condition necessary to access or complete learning materials, either here or as part of more formal university course evaluations. Since participation was voluntary, the evaluation may be subject to self-selection bias, consistent with limitations observed in course evaluation research (73).

Thirdly, the module feedback survey consisted of a relatively small number of questions. Although this evaluation approach was chosen based on consultation throughout the design process to limit participation burden, it is possible that not all strengths and concern related to AVA Online were captured by the surveys. This will be considered as AVA Online’s evaluation strategy continues to evolve.

Lastly, some examined outcomes, such as the promotion of relevant skills, require specific testing and longer-term evaluation to determine whether AVA Online actually resulted in sustained impact and behavioral change. This requires further research to assess more accurately.

## 5 Conclusion

The purpose of this process evaluation was to assess the first iteration of the AVA Online training program. Findings suggest that cross-sector digital training infrastructure can support shared learning and collaboration across academic and community contexts, while also revealing design tensions related to accessibility, usability, and perceived applicability. Despite these challenges, findings indicate that AVA Online is useful, applicable, and generally accessible, with several organizations already recognizing or incorporating AVA Online into training and professional development activities. Ongoing refinement reflects the iterative nature of sustainable knowledge-to-action processes, with continued emphasis on platform improvement and expansion into role-specific streams. AVA is also seeking funding to support broader global implementation in high-burden settings, alongside increasing global interest. AVA Online contributes to efforts to address and prevent GBV and ACEs by demonstrating how scalable, adaptable online training platforms may support timely and practice-oriented knowledge dissemination.

## Data Availability

The raw data supporting the conclusions of this article will be made available by the authors, without undue reservation.
